# Structure and transcription of the *Helicoverpa armigera densovirus* (HaDV2) genome and its expression strategy in LD652 cells

**DOI:** 10.1186/s12985-017-0691-y

**Published:** 2017-02-07

**Authors:** Pengjun Xu, Robert I. Graham, Kenneth Wilson, Kongming Wu

**Affiliations:** 1 State Key Laboratory for Biology of Plant Diseases and Insect Pests, Institute of Plant Protection, Chinese Academy of Agricultural Sciences, No. 2 West Yuan Ming Yuan Road, Beijing, 100193 People’s Republic of China; 2 Tobacco Research Institute, Chinese Academy of Agricultural Sciences, No. 11 Ke Yuan Jing Si Road, Qingdao, 266101 People’s Republic of China; 3 Crop and Environment Sciences, Harper Adams University, Newport, TF10 8NB UK; 4 Lancaster Environment Centre, Lancaster University, Lancaster, LA1 4YQ UK

**Keywords:** Densovirus, HaDV2, Expression, Transcription

## Abstract

**Background:**

Densoviruses (DVs) are highly pathogenic to their hosts. However, we previously reported a mutualistic DV (HaDV2). Very little was known about the characteristics of this virus, so herein we undertook a series of experiments to explore the molecular biology of HaDV2 further.

**Results:**

Phylogenetic analysis showed that HaDV2 was similar to members of the genus *Iteradensovirus*. However, compared to current members of the genus *Iteradensovirus*, the sequence identity of HaDV2 is less than 44% at the nucleotide-level, and lower than 36, 28 and 19% at the amino-acid-level of VP, NS1 and NS2 proteins, respectively. Moreover, NS1 and NS2 proteins from HaDV2 were smaller than those from other iteradensoviruses due to their shorter N-terminal sequences. Two transcripts of about 2.2 kb coding for the NS proteins and the VP proteins were identified by Northern Blot and RACE analysis. Using specific anti-NS1 and anti-NS2 antibodies, Western Blot analysis revealed a 78 kDa and a 48 kDa protein, respectively. Finally, the localization of both NS1 and NS2 proteins within the cell nucleus was determined by using Green Fluorescent Protein (GFP) labelling.

**Conclusion:**

The genome organization, terminal hairpin structure, transcription and expression strategies as well as the mutualistic relationship with its host, suggested that HaDV2 was a novel member of the genus *Iteradensovirus* within the subfamily *Densovirinae*.

**Electronic supplementary material:**

The online version of this article (doi:10.1186/s12985-017-0691-y) contains supplementary material, which is available to authorized users.

## Background

The subfamily of *Densovirinae* within the family *Parvoviridae* is a group of small (18–26 nm diameter), non-enveloped, icosahedral viruses containing a linear single-stranded DNA genome ranging between 4 and 6 kb with characteristic terminal hairpins [1–3]. Members of this subfamily typically produce “cellular dense nucleosis” pathogenesis in their hosts, hence, they are commonly termed densoviruses (DVs) [4–8]. Since the first identification of a densovirus in the greater wax moth *Galleria mellonella* [9], DVs have been isolated from many arthropods, including species from six insect orders (Lepidoptera, Diptera, Orthoptera, Dictyoptera, Odonata and Hemiptera) and decapod crustaceans (shrimps and crabs) [10–12].

To date, many DVs have been identified and sequenced. Unlike vertebrate parvoviruses, which all exhibit a monosense organization of their genome with nonstructural protein (NS) and structural protein (VP) open reading frames (ORFs) located on the same strand, arthropod DVs possess two types of genomes: monosense and ambisense [13–18]. Previously, the taxonomy of DVs was ambiguous, which was based on the organization of coding sequences, as well as genome size, terminal hairpin structure, gene expression strategy and host range [19]. Under the proposal of the International Committee on Taxonomy of Viruses (ICTV), Cotmore et al. [2] reconstructed the taxonomy of the family *Parvoviridae* in which DVs were classified into five distinct genera: *Ambidensovirus*, *Brevidensovirus, Iteradensovirus, Hepandensovirus* and *Penstyldensovirus* according to phylogenetic analysis and sequence homology.

DVs are highly pathogenic viruses to their hosts, and have been documented as being transmitted both horizontally and vertically [7, 9, 16]. Traditionally, these properties have captured the interest of many researchers investigating the potential application of DVs as biopesticides for biological control of insect pests or vectors for transgenic insects [20–26]. However, we previously reported a novel DV displaying a mutualistic interaction with its host (*Helicoverpa armigera*), and named this virus HaDV2 (previously named HaDNV-1) to distinguish it from the HaDV1 reported by El-Far et al. [27–29]. In this current study, we report the genome organization, transcription and expression strategies of the virus HaDV2.

## Methods

### Insect cell culture and transfection

*Lymantria dispar* LD652 cells, a gift from Central China Normal University in Wuhan (China) [30], were cultured in Grace’s insect medium containing 10% fetal bovine serum (FBS) and 1% Penicillin-Streptomycin (Invitrogen, Grand Island, NY, USA) at 28 °C. Purified plasmid (100 ng) containing the ORFs of NS1 and NS2 with TIANpure Mini Plasmid Kit (TIANGEN, Beijing, China) was transfected into cells using Cellfectin® II Reagent as recommended (Invitrogen). The luciferase activities were determined using Luciferase Assay System (Promega, Madison, WI, USA).

### Sequence analysis

Identity and alignment of the nucleotide and amino acid sequences was calculated using CLUSTAL W software [31]. The ORFs were identified using the ORF Finder (http://www.ncbi.nlm.nih.gov/orffinder/). Neighbor-joining trees with Poisson-corrected distances for the DV nucleotide sequences and the amino acid sequences of (NS1, NS2 and VP ORFs) were constructed using CLUSTAL W software and MEGA6.0 software [32].

### Amplification of the stem-loop structure of HaDV2 genome by inverse PCR

The viral DNA was extracted from purified virus particles using TIANamp Genomic DNA Kit (TIANGEN). According to the reported genome sequence of HaDV2 (GenBank accsession No.: HQ613271), three forward primers near the 3′ end (DVF1 [nt 4576–4595], DVF2 [nt 3891–3910], DVF3 [nt 4343–4362]), and two reverse primers near the 5′ end (DVR1 [nt 1038–1057] and DVR2 [nt 832–889]) were designed according to the genome sequence of HaDV2 (Additional file 1: Table S1). PCR reactions were performed using TransTaq DNA Polymerase High Fidelity (TransGen, Beijing, China) and extracted viral DNA as a template. The PCR program was as follows: 30 s at 94 °C, 30 s at 57 °C, and 60 s at 72 °C for 40 cycles.

### Mapping of the transcripts by 5′/3′ RACE and Northern blot

The 5′ and 3′ ends of the HaDV2 transcripts were amplified using the SMART RACE cDNA Amplification Kit (Clontech, CA, USA), according to the manufacturer’s instructions. cDNA was synthesized by RT-PCR from the total RNA of migrating cotton bollworms infected by HaDV2 using primers NS3F1/UPM for the 3′ end, NS5R1/UPM and NS5R2/UPM for the 5′ end of the NS genes, 3 F1/UPM for the 3′ end, VP5R1/UPM and VP5R2/UPM for the 5′ end of the VP genes, respectively (Additional file 1: Table S1). RNA (30 μg total) from insects infected by HaDV2 were separated on 1.1% formaldehyde agarose gels using MOPS buffer and blotted onto a positively charged nylon membrane (Roche, USA). Northern blot hybridization was performed using DIG-labeled probes (DIG DNA Labeling and Detection Kit, Roche, USA), according to the manufacturer’s instruction. A 543 bp NS probe (1073–1615 nt) and a 420 bp VP probe (4081–4500 nt) were amplified by PCR with primers pairs NSF/NSR and VPF/VPR (Additional file 1: Table S1) using 30 cycles on a thermocycler as follows: 30 s at 95 °C, 30 s at 50 °C, and 30 s at 72 °C.

### Antibody production

Using the predicted amino acid sequences gained from the earlier experiments in this study, two polypeptides were synthesized to raise polyclonal antisera in rabbits: CWDRAEFLRKYRKKVN and CDIGKSELWAPSVNPT for NS1 and NS2 proteins, respectively. The polypeptide of NS1, NS2 or VP was each emulsified with an equal volume of Freund’s complete adjuvant for the first injection and incomplete adjuvant for subsequent injections. Antisera were obtained by injecting an adult rabbit subcutaneously with 500 μg polypeptide, followed by three additional injections of 300 μg polypeptide at 20 days intervals. The serum was purified and stored at −70 °C. The titer of the antisera was measured using ELISA as described by Liu et al. [33].

### NS protein expression and subcellular localization

To characterize the expression of HaDV2 NS proteins in insect cells, we constructed two plasmids. Firstly, the HaDV2 NS promoter was amplified by primers NSPF/NSPR (Additional file 1: Table S1), digested with restriction endonuclease *Kpn*I/*Hind*II and cloned into a luciferase reporter vector pGL-3 Basic (Promega). This created the pNSP-Luc plasmid, in which the luciferase gene was under the control of the HaDV2 promoter. Secondly, the complete ORFs of HaDV2were amplified and cloned into the pEASY-T Cloning Vector (TransGen) to create pHaDNV-T with the primers HDVF1/HDVR1, HDVF2/HDVR2, HDVF3/HDVR3 (Additional file 1: Table S1), and using restriction endonucleases *Sac*II, *Afl*II and *Bsp*1407I, The resulting plasmid pHaDNV-T contained the whole genome of HaDV2except for the hairpin structure. LD652 cells transfected with pHaDNV-T plasmid were then analyzed using 12% SDS-PAGE and transferred onto PVDF membranes. The membranes were blocked with dry skimmed milk (5%) and incubated in PBST buffer (PBS containing 0.1% Tween-20) containing polyclonal antibodies (anti-VP, anti-NS1 or anti-NS2) (1:5000, 1 h) and then a horseradish peroxidase (HRP)-conjugated secondary antibody (ZSGB-BIO, China) (1:20000, 1 h). The blots were revealed using the Easysee Western Blot Kit (Transgen).

To investigate the subcellular localization of these proteins, the NS1 and NS2 ORFs were amplified with primers NS1LF/NS1LR and NS2LF/NS2LR (Additional file 1: Table S1), then cloned into plasmid pIE-Atg6-GFP [34] by exchanging BmAtg6 with these two ORFs to create NS1-GFP and NS2-GFP constructs, respectively. The constructs were then transfected into LD652 cells and the NS-GFP fusion proteins were examined by fluorescent microscopy 24 h post-transfection. The IE2-GFP plasmid which was constructed by inserting the OpIE2 promoter of the pIZ-V5/His (Invitrogen) into the plasmid pEGFP-N1 digested by restriction enzymes *Bgl* II and *Sac* I was used as control and obtained as a gift from Dr. Liu’s lab in Central China Normal University (China).

## Results

### Nucleotide sequences based analysis of HaDV2 genome organization

The size of virus particles of HaDV2 is about 20 nm in diameter and it possesses a monosence genome about 5 kb. The sequencing analysis of the HaDV2 genome reported in our previous study showed that it differed considerably from other known DVs (Additional file 1: Figure S1) [28]. HaDV2 contained three large ORFs on the same strand ORF1 (1260 nt in length) encoded the putative NS2 polypeptide of 419 amino acids with a predicted molecular mass of 48 kDa and a theoretical isoelectric point (pI) of 7.10. ORF2 (2010 nt in length) encoded the putative NS1 protein of 669 amino acids with a predicted molecular mass of 78 kDa and a pI of 5.87. Alignment of the HaDV2 NS1 amino acid sequence with three other iteradensoviruses indicated that HaDV2 NS1 protein shared two functional domains. Firstly, amino acids between aa 258–311 contained the two highly conserved replication initiator motifs involved in initiation and termination of rolling circle replication; and secondly, amino acids from aa 514–635 contained the NTP-binding and helicase domains typical of the NS1 polypeptide. Alignment analysis indicated that the amino acid sequences of NS1 and NS2 from the HaDV2 were shorter than those from closely related members of the genus *Iteradensovirus* (Additional file 1: Figure S2). ORF3 (1980 nt in length) encoded a putative VP protein of 659 amino acids with a molecular mass of 75 kDa and a pI of 7.13. The highly conserved phospholipase A2 domain located at amino acid positions aa 2–56 was also found in the VP ORF of HaDV2.

### Tree-based analysis

**Fig. 1:**
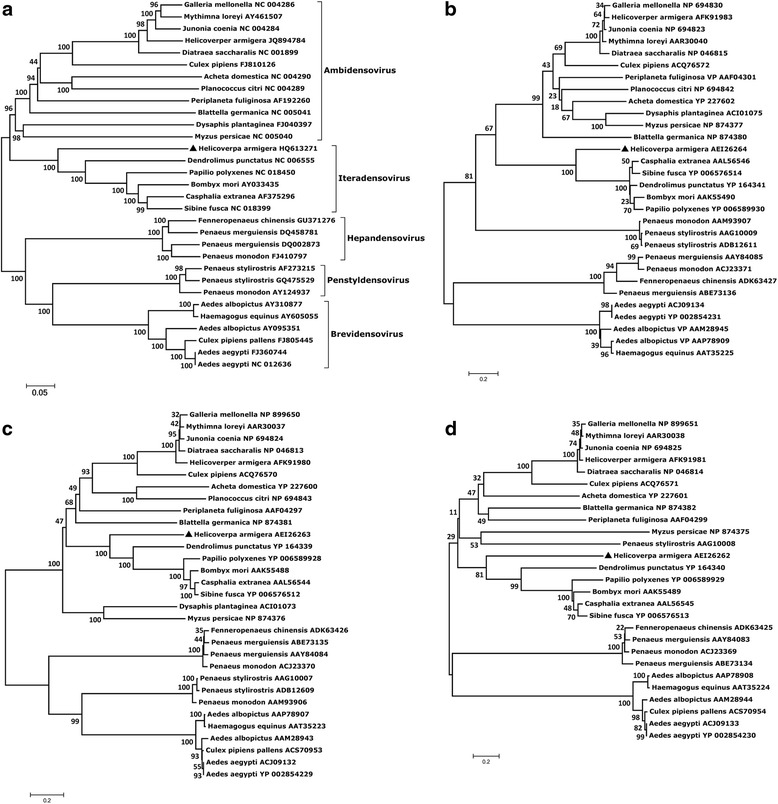
Neighbor-joining phylogenetic trees for members of the densoviruses, including **a** the genomic sequence, **b** the amino acid sequence of the VP ORF, **c** the amino acid sequence of the NS1 ORF, and **d** the amino acid sequence of the NS2 ORF. Other accession numbers are given after the abbreviated names. ▲ = HaDV2. Bootstrap values (1000 pseudoreplicates) > 50% are indicated on the nodes

Neighbor-joining trees were constructed using the whole genome sequence of HaDV2 as well as the putative amino acid sequences of NS1, NS2 and VP ORFs. Thirty-one DVs from other arthropods that had either the complete genome or the full coding sequence were included in the analysis (Fig. 1). The trees revealed five main branches: branch 1 included all known DV members with an ambisense genome in the genus *Ambidensovirus*; branch 2 included members of the genus *Iteradensovirus* and HaDV2; branch 3 included members of the genus *Hepandensovirus*; branch 4 included members of the genus *Penstyldensovirus*; and branch 5 consisted of members of the genus *Brevidensovirus* (Fig. 1a). The branches of the tree constructed with amino acid sequences of VP was similar to that of the nucleotide sequence tree (Fig. 1b). The trees constructed with amino acid sequences of NS1 and NS2 differed considerably from the trees described above (see Fig. 1c, d). Although the four trees were not identical, they all indicated that the HaDV2 was most closely related to the members of the genus *Iteradensovirus*. We also reconstructed the trees using Maximum likelihood method and the trees showed similar topology with the NJ trees except for NS2-tree (Additional file 1: Figure S3). Alignment of the nucleotide sequence and amino acid sequences (of VP, NS1 and NS2) indicated that sequence identities between viruses within the genus *Iteradensovirus* exceed 58, 71, 35 and 28%, respectively; and that the identities between HaDV2 and members of the genus *Iteradensovirus* are no more than 44, 36, 28 and 19%, respectively.

### Determination of the stem-loop structure of HaDV2 genome by inverse PCR

**Fig. 2:**
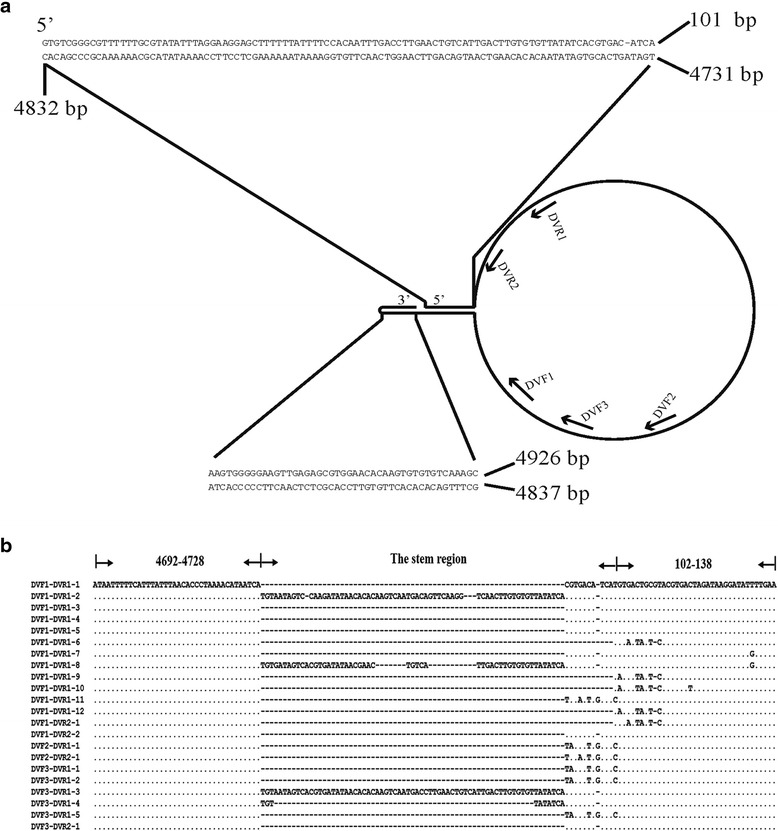
Determination of the stem-loop structure with the HaDV2 sequence. **a** The predicted stem-loop structure of HaDV2 genome. Nucleotides of the 3′ terminal hairpin and reverse complementary sequences are shown. Numbers indicate locations of the nucleotides on the HaDV2 genome (5′-3′). The primers used for determining the stem-loop structure were shown, including three forward primers DVF1, DVF2, DVF3 and two reverse primers DVR1, DVR2. **b** Alignment of terminal nucleotide sequences from different clones to confirm the stem-loop structure. Clone names shown on the left indicate their amplification primers and clone numbers (e.g., clone DVF1-DVR1-1 stands for one clone amplified by primers DVF1/DVR1). The numbers show the nucleotide location on the genome of HaDV2. “The stem region” indicates the double DNA region as show in (**a**). “.” = identical nucleotides; “-” = absence of nucleotides

Sequence analysis indicated that the HaDV2 terminal-ends could form a stem-loop structure by a reverse complementary sequence (ITRs) near the two ends (Fig. 2a). To further confirm this prediction, PCR successfully amplified segments with forward primers near the 3′ end and reverse primers near the 5′ end. Sequence alignment indicated that all the amplified sequences were consistent with those of the HaDV2 except for the stem region located at ITRs (Fig. 2b).

### Transcript analysis of HaDV2

**Fig. 3:**
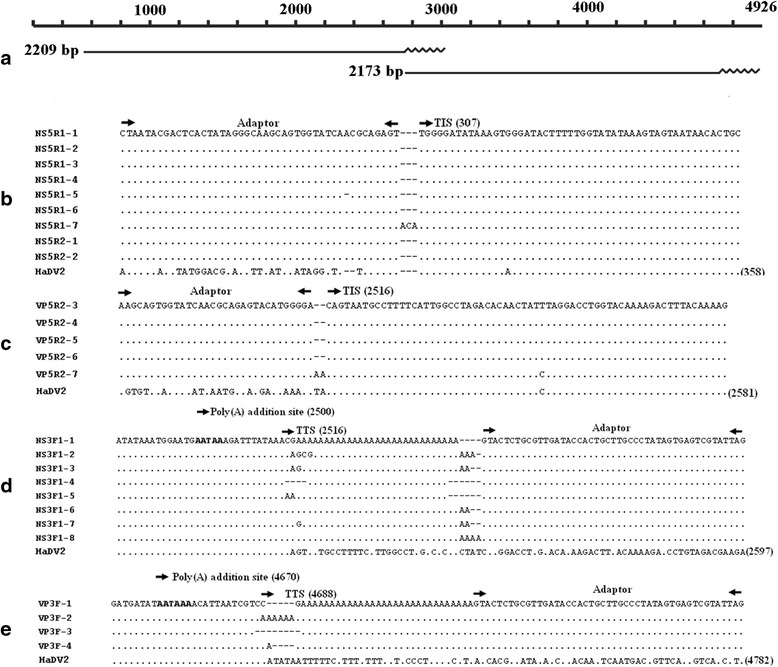
Transcriptional analysis of HaDV2 by RACE. **a** The putative HaDV2 transcripts were shown according to the results of 3′ and 5′ RACE. The length of each transcript is indicated on the *left* except for the nucleotides of poly(A) (*wavy lines*). **b** and **c** Analysis of the transcriptional initiation site (TIS) by 5′ RACE with NS and VP primers, respectively. **d**, **e** Analysis of the transcriptional terminal site (TTS). The TIS, TTS and adaptor sequence are indicated by *arrows*. The number of TISs, TTSs and the consensus sequence are shown (HaDV2). “.” = identical nucleotides; “-” = absence of nucleotides

**Fig. 4:**
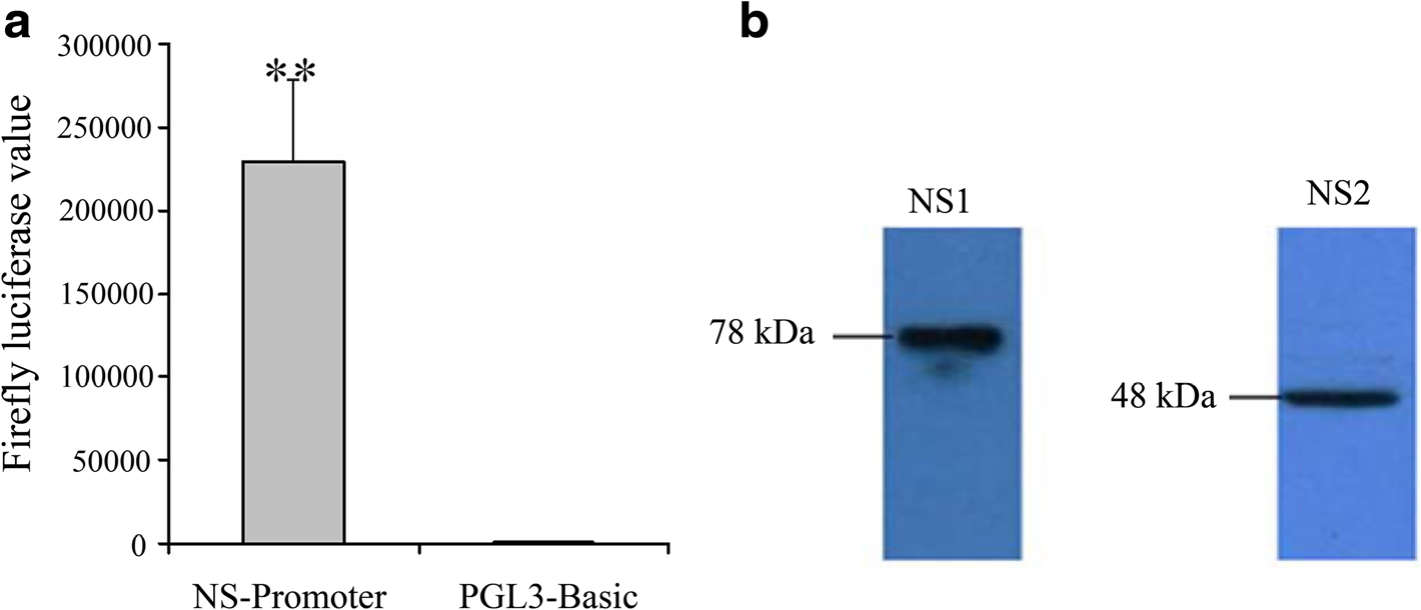
The expression of NS proteins in LD652 cells. **a** Analysis of the transcriptional activity of the NS promoter of HaDV2 in LD652 cells. **b** Western blot analysis of the proteins expressed by HaDV2 with anti-NS1 and anti- NS2 antibodies using LD652 cells transfected with the pHaDNV-T plasmid

Using 5′ and 3′ RACE primers, two transcription initiation sites (TISs) (at positions nt 307 (Fig. 3b) and nt 2516 (Fig. 3c)) and two transcription termination sites (TTSs) (at positions nt 2516 (Fig. 3d) and nt 4688 (Fig. 3e)) were determined. The TTS for the NS genes occurred at nt 2516, with canonical poly(A) addition sites (AATAAA) located 16 nucleotides upstream from the TTS (Fig. 4d). The VP TTS was located at nt 4688, with canonical poly(A) addition sites situated 18 nucleotides upstream from the TTS (Fig. 4e). The transcript of 2209 bp ending at nt 2516 may encode the NS1 and NS2 proteins. The transcripts of 2173 bp ending at nt 4688 may encode the VP proteins (Fig. 4a).

Northern Blot analysis was undertaken to determine the size and relative abundance of the transcripts for each of the three viral ORFs. Hybridization of total RNA isolated from infected insects revealed one band of 2.2 kb when using the NS probe; and one band of 2.2 kb when using the VP probe (Additional file 1: Figure S4a, b).

### The expression and subcellular localization of NS1 and NS2 proteins of HaDV2 in LD652 cells

**Fig. 5:**
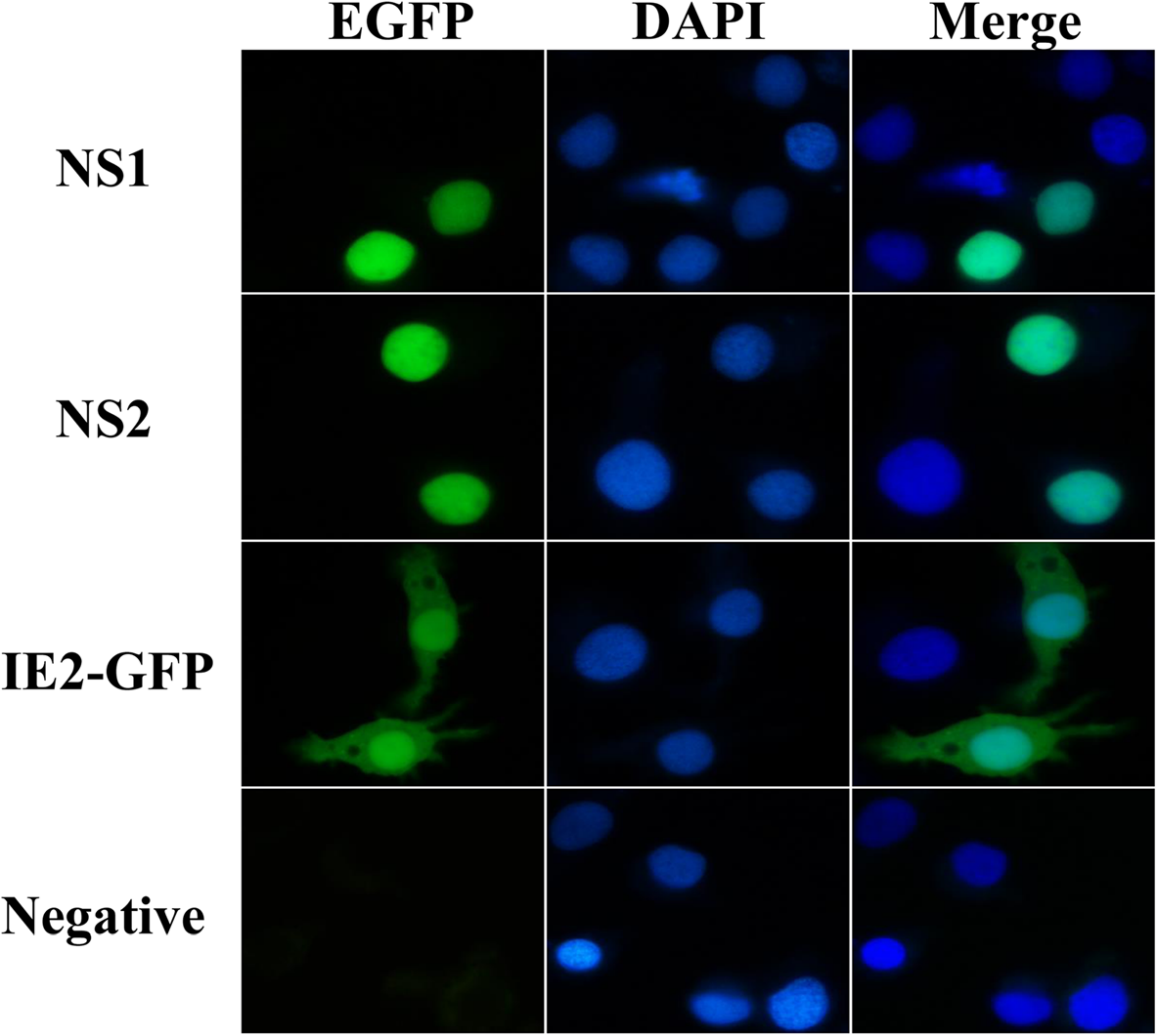
Subcellular localization of IE2-GFP, NS1-GFP and NS2-GFP fusion proteins in LD652 cells (×400), indicating the proteins of NS1 and NS2 were located to the nucleus

The expression of HaDV2 NS proteins was undertaken in *Lymantria dispar* LD652 cells. To determine the functionality of the NS promoter, the NS promoter-luciferase construct pNSP-Luc was transfected into LD652 cells and lucifarase activity was measured 24 h post-transfection. Our results showed that the luciferase activity driven by the NS promoter was approximately 225 times higher than that of the promoterless control vector pGL3-Basic (Fig. 4a), indicating that the transcription machinery of the LD652 cells recognized the NS promoter leading to expression of HaDV2 NS proteins. Western blot analyses of pHaDV-T transfected LD652 cells using antisera prepared against NS1 and NS2 proteins (Additional file 1: Figure S5) revealed two proteins of 78 kDa (NS1) and 48 kDa (NS2) (Fig. 4b). Both the NS1 and NS2 GFP-fused proteins were exclusively present within the nucleus of the host cells, suggesting NS1 and NS2 might localize within the nucleus (Fig. 5).

## Discussion

DVs are a group of viruses usually associated with causing high pathogenicity to their hosts [7, 9, 12]. However, we previously reported a novel DV (HaDV2) which was found to be beneficial to its host by increasing larval and pupal developmental rate, fertility, adult female lifespan and enhancing host resistance to both a baculovirus and low doses of the Bt toxin [28, 29]. This suggested a virus with quite different characteristics to the other previously described members within subfamily Densovirinae. In this current study, we determined the molecular biology of the HaDV2 virus, namely through examining its genome structure and ORF transcription and expression strategy. Based on our results, HaDV2 was a novel member of genus *Iteradensovirus*, with new features differing from other members from this genus, such as an ITR of 101 nt at both termini, a single 90 nts hairpin structure at the 3′ end and the first ORF encoding NS2 protein [17, 28, 35–40].

Phylogenetic analysis using both nucleotide and amino acid sequences showed that HaDV2 was clustered within the genus *Iteradensovirus*. The sequence identities of the viral DNA and the amino acid identities for VP, NS1 and NS2 ORFs among members of the genus *Iteradensovirus* exceed 58, 71, 35 and 28%, respectively. However, the sequence identities between HaDV2 and the current members of the genus *Iteradensovirus* are no more than 44, 36, 28 and 19%, respectively. Thus, although the HaDV2 was clustered with *Iteradensovirus*, it differs considerably from the other iteradensoviruses and appears to have a different function as described previously [29].

Although phylogenetic analysis indicated that HaDV2 was clustered with members of the genus *Iteradensovirus*, the NS1 and NS2 proteins of the HaDV2 are smaller than those of other *Iteradensovirus* (more than 753 and 451 amino acids, respectively) [17, 28, 35–40]. Are they functionally expressed as predicted? We used Western Blot analysis of transfected LD652 cells using anti-NS1 and anti-NS2 to show that the NS1 and NS2 proteins were 78 kDa and 48 kDa, respectively, consistent with the predicted size of the protein. NS proteins are a pivotal factor for viral transcription and replication as well as pathogenicity. The replication of DVs occured in the nucleus of their hosts [19]. Therefore, the NS proteins of DVs should be located in the nucleus by nuclear localization signal (NLS) as reported by Yu et al. [40]. To further investigate whether the NS proteins of the HaDV2 localized within the nucleus of their hosts (as those of other DVs), NS1 and NS2 proteins were expressed in LD652 cells using the recombinant plasmid NS1-GFP and NS2-GFP. The result indicated that the NS1 and NS2 proteins were completely located in the nucleus, suggesting that they possess a common function and could possibly play a role in the novel interactions between HaDV2 and its host. The experiments with the NS proteins were carried out by transient expression in LD652 cells, which were not the virus’s original host. It is acknowledged that this expression may not reflect the real role of HaDV2-NS promoter and how it works in the natural host.

Transcriptional patterns are diverse among the DVs. For example, JcDV, *Galleria mellonella densovirus* (GmDV) and *Mythimna loreyi densovirus* (MlDV) all have one transcript for the VP gene and two transcripts for the NS genes (the larger one for NS1 and the smaller one for NS2), in which the ORFs of NS1 and NS2 share a common TTS [15, 30, 41]. Meanwhile, the transcripts of CpDV, *Periplaneta fuliginosa densovirus* (PfDV) and *Myzus persicae nicotianae densovirus* (MpnDV) arise from alternative splicing [13, 42, 43]. The first ORFs of all known iteradensoviruses encode NS1 protein and the ORFs of NS2 are completely included in the ORFs of NS1 [40]. However, the first ORF of HaDV2 encodes NS2, which may impact gene expression of NS2 compared to NS1. In addition, the NS1 and NS2 of other iteradensoviruses were translated from different transcripts and the TIS of NS1 was found to start 2–26 nt upstream of the start codon [40]. Unexpectedly, our results suggested that the NS1 and NS2 of HaDV2 translated from the same transcript which started 63 nt upstream of the start codon of NS2. Surprisingly, although we provide evidence of the activity of the NS promoter, we failed to find the TATA-box upstream of the TIS of NS. Two TATA-box like sequences were located at nts 313 and 335 upstream of the start codon of NS1 and NS2; suggesting HaDV2, maybe like brevidensoviruses, has overlapping NS gene promoters responsible for different transcript starts and dictating the relative transcription rates of these transcripts. However, one of the two transcripts was in great excess, making it difficult to detect both transcripts by RACE. Like other DVs, the VP transcripts of HaDV2 had short-untranslated regions, located at 5 nts upstream of the start codon of VP.

## Conclusion

We report a novel densovirus, assigned as HaDV2, which differs from the other DVs in its genome organization, terminal hairpin structure, and transcription and expression strategies. Taken together with the unique mutualistic relationship previously described between HaDV2 and its host [29], this strongly indicates that HaDV2 is a novel member within the genus *Iteradensovirus*.

## Additional file

Additional file 1: Table S1. Primers used in this study. **Figure S1.** The virus particles and its genome organization. (a) Electron micrograph of HaDNV-1 viruses purified from adult *Helicoverpa armigera* negatively stained with uranyl acetate (×200000). Bar, 100 nm. (b) Agarose gel electrophoresis (1%) of the extracted HaDNV-1 DNA. Lane 1 = DNA from the HaDNV-1, Lane 2 = Marker. (c) The putative ORFs of HaDNV-1. The plus strand contains three large ORFs: ORF1, ORF2 and ORF3, which encode NS2, NS1 and VP proteins, respectively. (d) Hairpin structure in the 3′ terminus of HaDNV-1 predicted by the QuickFOLD program. The numbers in bracket stand for the start and stop nucleotides of the hairpin on the HaDNV-1 genome. **Figure S2.** Alignment of amino acid sequences of NS1 (a) and NS2 (b) of HaDNV-1 with the ones of members from *Iteravirusdensovirus*. HaDNV = *Helicoverpa armiger*a densovirus 1 (accession number: HQ613271), BmDNV = *Bombyx mori* densovirus 1 (AY033435), CeDNV = *Casphalia extranea* densovirus (AF375296), DpDNV = *Dendrolimus punctatus* densovirus (NC_006555). **Figure S3.** The maximum-likelihood tree for members of the densoviruses, including (a) the genomic sequence with GTR + G + I model, (b) the amino acid sequence of the VP ORF with LG + G model, (c) the amino acid sequence of the NS1 ORF with LG + G + I model, and (d) the amino acid sequence of the NS2 ORF with JTT + G model. “▲” represents the sequence of HaDV2. Bootstrap values (1000 pseudoreplicates) > 50% are indicated on the nodes. **Figure S4.** Northern blot analysis of the HaDV2 transcripts showed two bands of 2.2 kb with the NS and the VP probe, respectively. **Figure S5.** Dose-responses of anti-NS1, anti-NS2 and anti-VP antibodies using ELISA. (DOC 2203 kb)

## Abbreviations

DV: Densovirus
ICTV: The international committee on taxonomy of viruses
NS: Nonstructural protein
ORF: Open reading frame
pI: Theoretical isoelectric point
VP: Structural protein
