# Supplementary material for: Structure and transcription of the Helicoverpa armigera densovirus (HaDV2) genome and its expression strategy in LD652 cells
Source: Virol J. 2017 Feb 7;14:23. doi: 10.1186/s12985-017-0691-y (PMC5296992; doi:10.1186/s12985-017-0691-y)
Supplement: Additional file 1: Table S1. — Primers used in this study. Figure S1. The virus particles and its genome organization. (a) Electron micrograph of HaDNV-1 viruses purified from adult Helicoverpa armigera negatively stained with uranyl acetate (×200000). Bar, 100 nm. (b) Agarose gel electrophoresis (1%) of the extracted HaDNV-1 DNA. Lane 1 = DNA from the HaDNV-1, Lane 2 = Marker. (c) The putative ORFs of HaDNV-1. The plus strand contains three large ORFs: ORF1, ORF2 and ORF3, which encode NS2, NS1 and VP proteins, respectively. (d) Hairpin structure in the 3′ terminus of HaDNV-1 predicted by the QuickFOLD program. The numbers in bracket stand for the start and stop nucleotides of the hairpin on the HaDNV-1 genome. Figure S2. Alignment of amino acid sequences of NS1 (a) and NS2 (b) of HaDNV-1 with the ones of members from Iteravirusdensovirus. HaDNV = Helicoverpa armigera densovirus 1 (accession number: HQ613271), BmDNV = Bombyx mori densovirus 1 (AY033435), CeDNV = Casphalia extranea densovirus (AF375296), DpDNV = Dendrolimus punctatus densovirus (NC_006555). Figure S3. The maximum-likelihood tree for members of the densoviruses, including (a) the genomic sequence with GTR + G + I model, (b) the amino acid sequence of the VP ORF with LG + G model, (c) the amino acid sequence of the NS1 ORF with LG + G + I model, and (d) the amino acid sequence of the NS2 ORF with JTT + G model. “▲” represents the sequence of HaDV2. Bootstrap values (1000 pseudoreplicates) > 50% are indicated on the nodes. Figure S4. Northern blot analysis of the HaDV2 transcripts showed two bands of 2.2 kb with the NS and the VP probe, respectively. Figure S5. Dose-responses of anti-NS1, anti-NS2 and anti-VP antibodies using ELISA. (DOC 2203 kb) [file 12985_2017_691_MOESM1_ESM.doc]

**Table S1.** Primers used in this study

| Primer name | Primer sequence (5'-3') | Location (nt) | Instruction |
| --- | --- | --- | --- |
| DVF1 | ATAATTGGGATATACCCGGT | 4576-4595 | Detection of the stem-loop structure |
| DVF2 | AGCGGTAAGAGATTGGTGTC | 3891-3910 |
| DVF3 | CGGAAGGAAAAAACCTACTG | 4343-4362 |
| DVR1 | GACTGCCATCCTGAGAACTT | 1038-1057 |
| DVR2 | CCGTGGTCCCCTTCTGTA | 872-889 |
| NSPF | GCGGTACCGTGTCGGGCGTTTTTTGC | 1-16 (with Kpn I site) | Amplification of the NS promoter |
| NSPR | GGAAGCTTAATACAACCGTCTTCTAAAC | 361-380 (with Hind III site) |
| HDVF1 | CCGCGGGTGTCGGGCGTTTTTTGC | 1-16 (with Sac II site) | Constructing the plasmid of pHaDNV-T |
| HDVR1 | TCGTCCTGCTTCATCGTGTGTG | 1109-1130 |
| HDVF2 | CCGCGGTTTACAGAAGGGGACCACGG | 870-889 (with Sac II site) |
| HDVR2 | TTCCATTCTGAACCCCAACATT | 3482-3503 |
| HDVF3 | CGGACAATGCTGGTGAGGC | 2969-2987 |
| HDVR3 | GTGTCGGGCGTTTTTTGC | 4815-4832 |
| NS1LF-GFP | GCGGTACCATGGAATCACGTGATC | 447-462 (with Kpn I site) | Constructing the plasmid of NS1-GFP |
| NS1LR-GFP | GCGGATCCGCAAGTTCATATTTAACATAG | 2435-2453 (with BamH I) |
| NS2LF-GFP | GCGGTACCATGGTTGTATTCTCTACT | 370-387 (with Kpn I site) | Constructing the plasmid of NS2-GFP |
| NS2LR-GFP | GCGGATCCAAATAAGTTTTCCGTTG | 1612-1627 (with BamH I) |
| NSF | GGGACTATTACCAAAGCCAG | 1073-1092 | Amplification of the NS probe using in norther blot |
| NSR | GTTGATATGTTTCTCTCTCCG | 1595-1615 |
| VPF | ATGGTAGAATGCTCAATC | 4081-4098 | Amplification of the VP probe using in norther blot |
| VPR | CTAGTTTTTTAATGCTTTTAC | 4480-4500 |
| NS3F1 | CGCCGATAGCCAAGGGAATCATGC | 1532-1555 | Amplification of the TTS site for NS genes |
| NS5R1 | ACCAGTGCTGTCTTCCACCTTTCGCTC | 1476-1503 | Amplification of the TIS site for NS genes |
| NS5R2 | AGACACACACGATGAAGCAGGACGA | 1106-1130 |
| VP5R1 | TTTTCTGCTGCTTCGGCGTCATCA | 4374-4397 | Amplification of the TIS site for VP genes |
| VP5R2 | TGTTTAGGTCTATGTGTCATTTCCCAGGCT | 3709-3738 |
| 3F1 | CGGACAAAGAGGATTCGTTACACGAGAC | 4119-4146 | Amplification of the TTS site for VP genes |


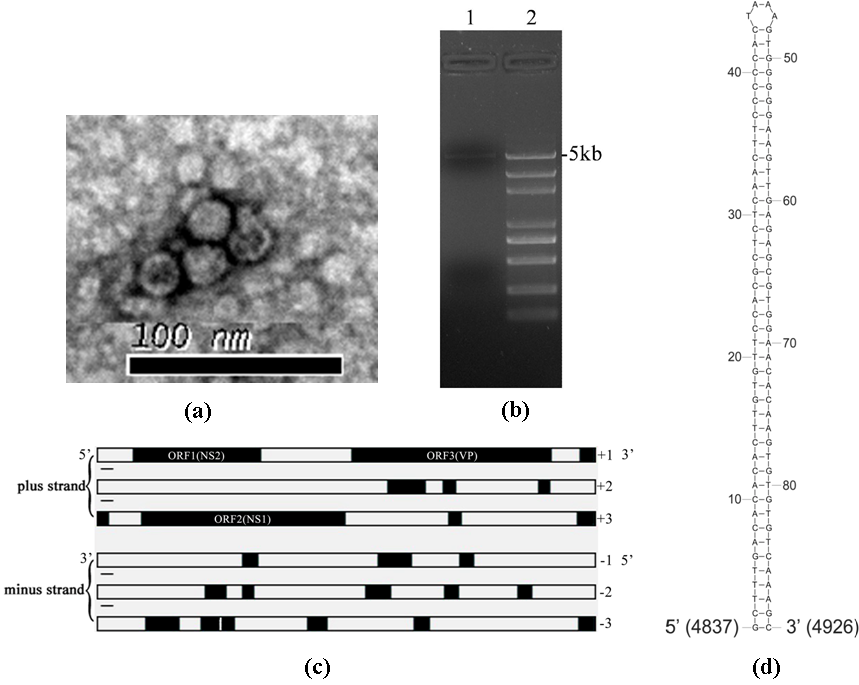


**Fig. S1** The virus particles and its genome organization. (a) Electron micrograph of HaDNV-1 viruses purified from adult *Helicoverpa armigera* negatively stained with uranyl acetate (×200000). Bar, 100nm. (b) Agarose gel electrophoresis (1%) of the extracted HaDNV-1 DNA. Lane 1=DNA from the HaDNV-1, Lane 2= Marker. (c) The putative ORFs of HaDNV-1. The plus strand contains three large ORFs: ORF1, ORF2 and ORF3, which encode NS2, NS1 and VP proteins, respectively. (d) Hairpin structure in the 3’ terminus of HaDNV-1 predicted by the QuickFOLD program. The numbers in bracket stand for the start and stop nucleotides of the hairpin on the HaDNV-1 genome.


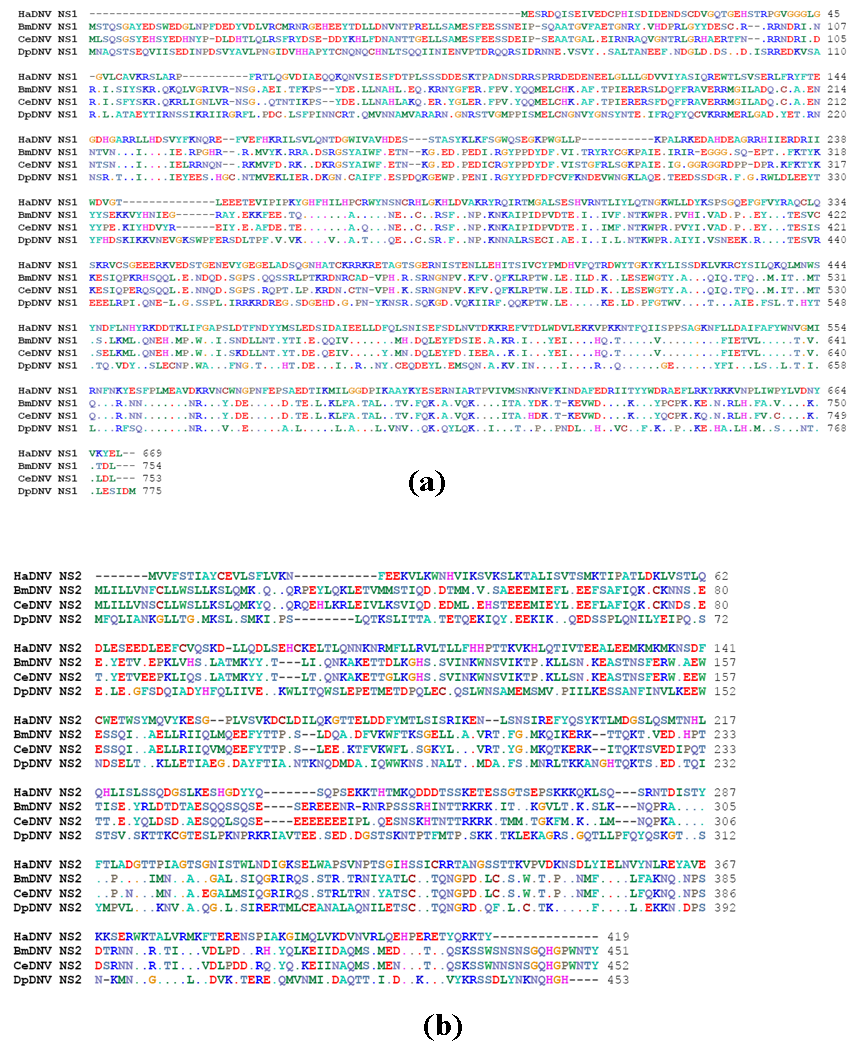


**Fig. S2** Alignment of amino acid sequences of NS1 (a) and NS2 (b) of HaDNV-1 with the ones of members from *Iteravirusdensovirus*. HaDNV=*Helicoverpa armiger*a densovirus 1 (accession number: HQ613271), BmDNV=*Bombyx mori* densovirus 1 (AY033435), CeDNV= *Casphalia extranea* densovirus (AF375296), DpDNV= *Dendrolimus punctatus* densovirus (NC_006555).


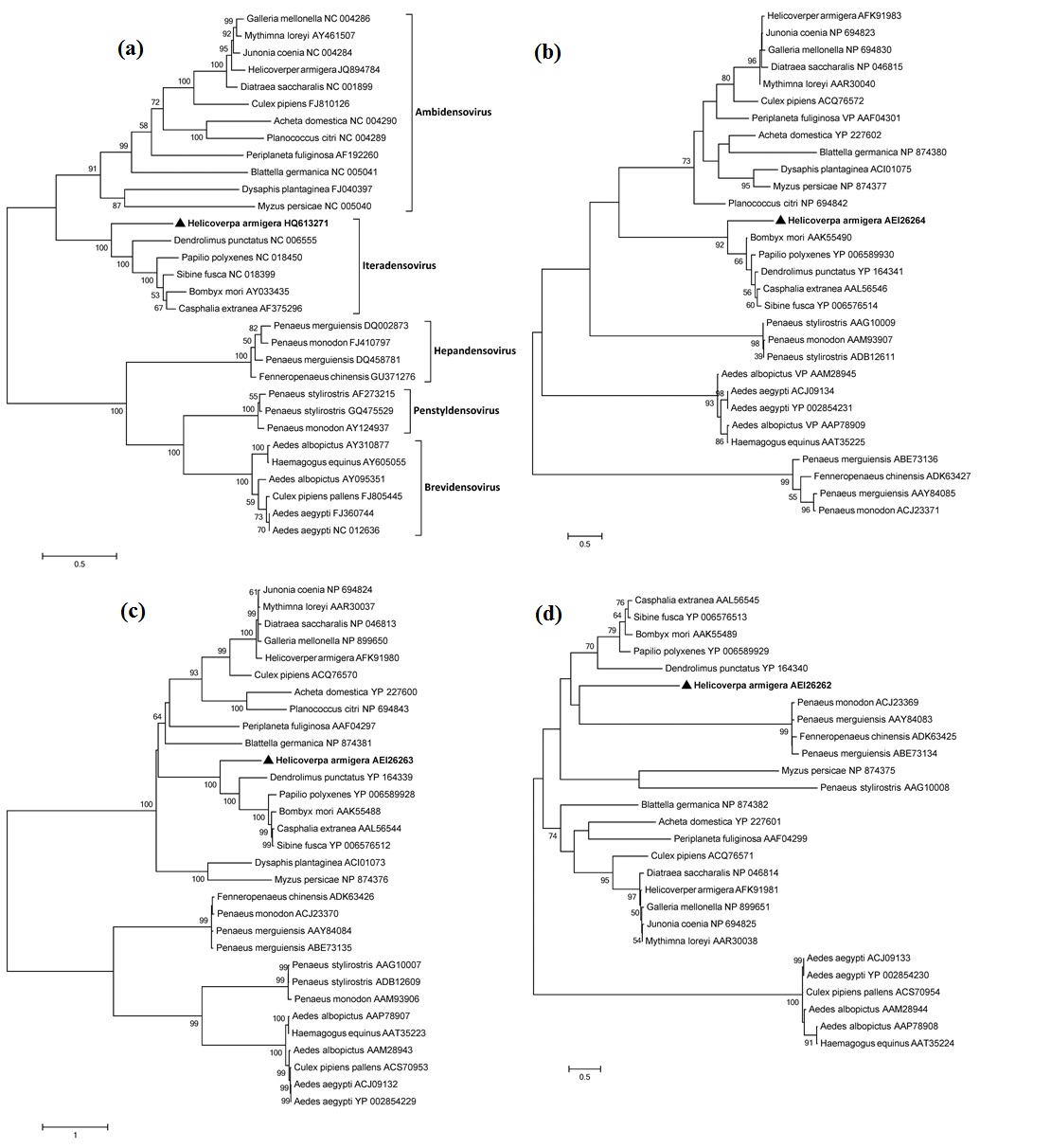


**Fig. S3** The maximum-likelihood tree for members of the densoviruses, including (a) the genomic sequence with GTR+G+I model, (b) the amino acid sequence of the VP ORF with LG+G model, (c) the amino acid sequence of the NS1 ORF with LG+G+I model, and (d) the amino acid sequence of the NS2 ORF with JTT+G model. “▲” represents the sequence of HaDV2. Bootstrap values (1000 pseudoreplicates) > 50% are indicated on the nodes.


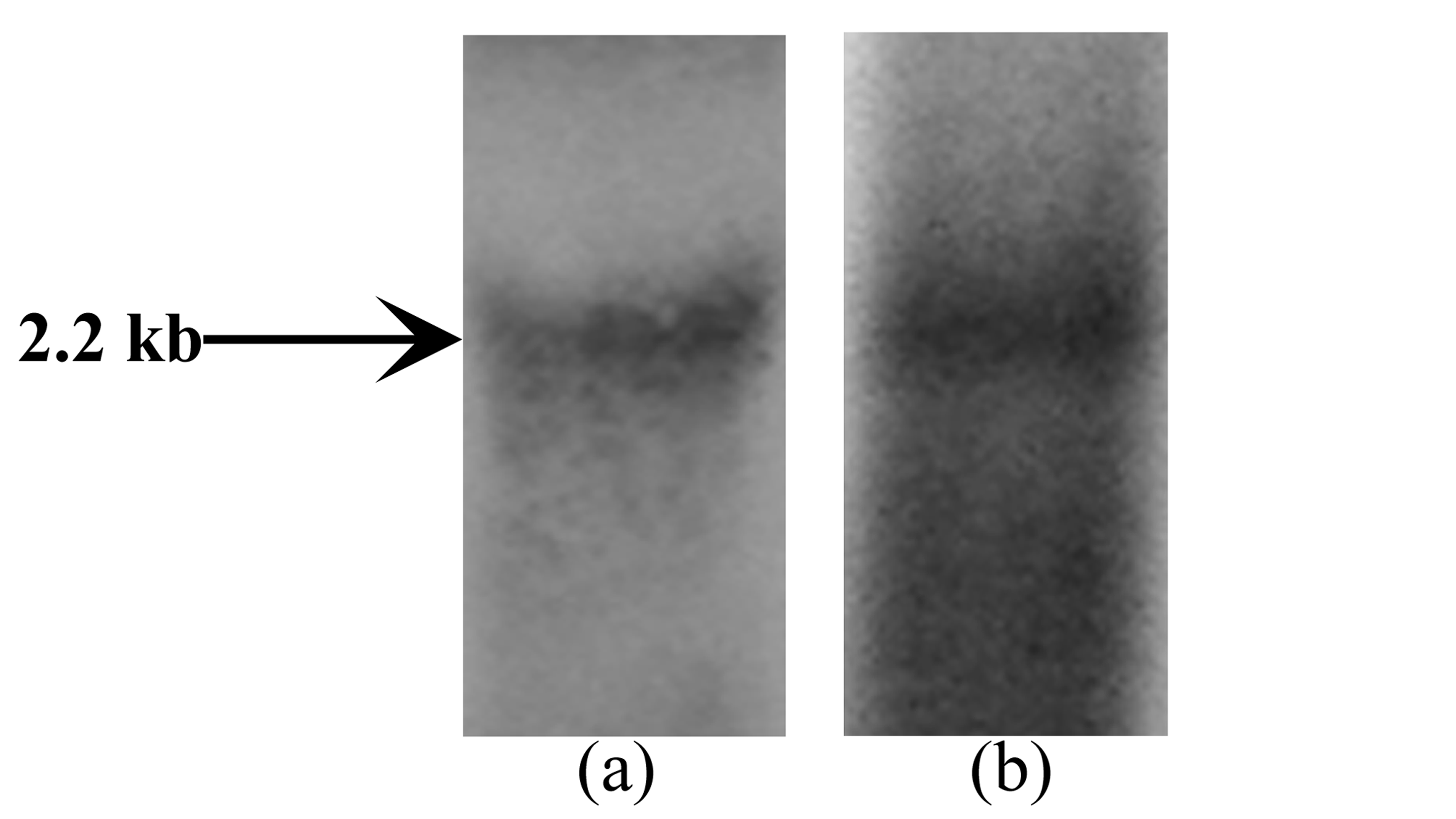


**Fig. S4** Northern blot analysis of the HaDV2 transcripts showed two bands of 2.2 kb with the NS and the VP probe, respectively.


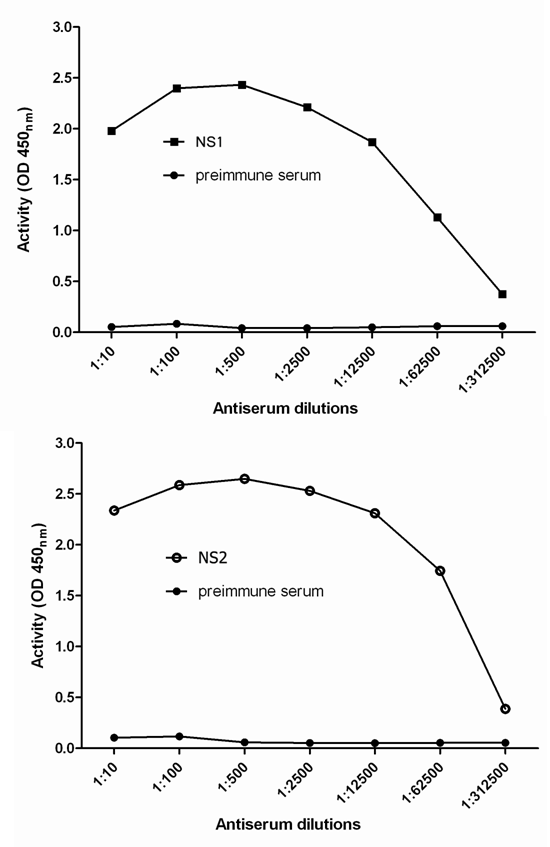


**Fig. S5** Dose-responses of anti-NS1, anti-NS2 and anti-VP antibodies using ELISA.
